# Comparative Analysis of Epigallocatechin-3-Gallate and TNF-Alpha Inhibitors in Mitigating Cisplatin-Induced Pancreatic Damage Through Oxidative Stress and Apoptosis Pathways

**DOI:** 10.1007/s12011-024-04239-9

**Published:** 2024-05-22

**Authors:** Enver Ciftel, Filiz Mercantepe, Tolga Mercantepe, Kerimali Akyildiz, Adnan Yilmaz, Serpil Ciftel

**Affiliations:** 1 Department of Endocrinology and Metabolism, Sivas Numune Hospital, Sivas, Turkey; 2 Department of Endocrinology and Metabolism, Faculty of Medicine, Recep Tayyip Erdoğan University, Rize, 53010 Turkey; 3 Department of Histology and Embryology, Faculty of Medicine, Recep Tayyip Erdoğan University, Rize, Turkey; 4 Department of Biochemistry, Faculty of Medicine, Recep Tayyip Erdoğan University, Rize, Turkey; 5 Department of Endocrinology and Metabolism, Erzurum Education and Research Hospital, Erzurum, Turkey

**Keywords:** Catechins, Cisplatin, Infliximab, Pancreas, Rat, TNF-α

## Abstract

Oxidative stress and inflammation caused by cisplatin, which is frequently used in the treatment of many cancers, damage healthy tissues as well as cancer cells. In this study, we aimed to investigate the effect of epigallocatechin-3-gallate (EGCG) and infliximab (INF) administration on pancreatic endocrine cells in rats treated with systemic cisplatin (CDDP). The rats were randomly divided into 6 groups: group 1 (control group), group 2 (EGCG group), group 3 (CDDP group), group 4 (EGCG + CDDP group), group 5 (CDDP + INF group), and group 6 (EGCG + CDDP + INF group). The study’s findings demonstrated that EGCG and INF effectively reduced the cellular damage induced by CDDP in histopathologic investigations of the pancreas. EGCG and INF, whether used individually or in combination, demonstrated a significant reduction in malondialdehyde (MDA) levels and an increase in glutathione (GSH) levels in the rat pancreas compared to the CDDP group. Immunohistochemically, the enhanced presence of insulin and glucagon positivity in the EGCG and INF groups, along with the absence of TUNEL immunopositivity, indicate that both treatments reduced CDDP-induced apoptosis. Furthermore, the observed lack of immunopositivity in TNF-α and 8-OHdG in the groups treated with EGCG and INF, compared to those treated with CDDP, indicates that these substances can inhibit inflammation. EGCG and INF, whether provided alone or together, can potentially reduce the damage caused to pancreatic islet cells by cisplatin. This effect is achieved through their anti-inflammatory and antioxidant properties during the early stages of the condition.

## Introduction

Cisplatin (cis-diamminedichloroplatinum, CDDP), a platinum-based antineoplastic agent, is used as a single agent or in combination therapy in the treatment of many cancers due to its high efficacy and proven effects on local control and survival [1]. As with numerous antineoplastic drugs, the clinical use of cisplatin (CDDP) is limited due to its toxic effects on multiple organs [2]. CDDP has been extensively investigated in various studies in the literature due to its widespread use and its association with multiple organ toxicities [3]. Especially in the acute period, CDDP-induced toxicity in the kidney, liver, and neural system represents an important handicap [4–6]. Although the pancreas is among the organs that exhibit CDDP-induced toxicity, it has received relatively less attention. However, studies exist indicating CDDP-induced pancreatic injury [7, 8]. Studies have reported a direct cytotoxic effect of CDDP on both the exocrine and endocrine compartments of the pancreas [9]. Moreover, the higher rates of hyperglycemia in individuals exposed to CDDP therapy relative to controls have been attributed to pancreatic toxicity [10]. The severity of this pancreatic injury is often characterized by increased oxidative stress and activation of apoptosis pathways [8, 9].

The effect mechanism of CDDP causes DNA damage and oxidative stress by impairing DNA repair through crosslinking with purine bases in the DNA, inducing cell apoptosis as a result [1]. In recent years, great importance has been given to identifying new therapeutic interventions to alleviate organ damage caused by cisplatin [11]. In the literature, various pharmacological and non-pharmacological agents have been investigated to prevent CDDP toxicity in healthy tissues [3]. Two promising candidates that show potential for clinical expansion are studies of epigallocatechin-3-gallate (EGCG), a polyphenolic organic found abundantly in white and green tea, and tumor necrosis factor-alpha (TNF-α) [12, 13]. It is thought that both agents may play a potential protective role in organ damage due to various causes by exhibiting anti-inflammatory and antioxidant properties.

TNF-α is a proinflammatory cytokine that accelerates cell damage and apoptosis, and its production is triggered by conditions of oxidative stress [14]. TNF-α is involved in the development and progression of various autoimmune disorders [15]. It is also thought to play a role in the development of autoimmune diabetes by causing beta-cell toxicity [16]. Infliximab is a monoclonal TNF-α inhibitor that is used in the treatment of several TNF-α-mediated disorders such as rheumatoid arthritis, psoriasis, ulcerative colitis, and Crohn’s disease [17–19].

EGCG is a polyphenol with a low side effect profile and proven safety that is found most abundantly in green tea [20]. EGCG is known to offer protection against DNA damage by scavenging reactive oxygen species (ROS), showing an antioxidant and anti-inflammatory effect [21]. EGCG also reduces the expression of proinflammatory mediators such as TNF-α. Data from the literature also indicate that EGCG inhibits the growth of cancer cells and induces their apoptosis [13, 22, 23].

The primary objective of this study is to assess and compare the efficacy of EGCG and TNF-alpha inhibitors in mitigating pancreatic injury induced by cisplatin. While serving as an antineoplastic drug, cisplatin might cause detrimental effects on pancreatic tissues, potentially through the mechanisms of oxidative stress and apoptosis. Hence, our objective is to conduct comparative research in order to assess the capacity of EGCG and TNF-alpha inhibitors to hinder or mitigate pancreatic harm caused by cisplatin. Additionally, we seek to comprehend the mechanisms that drive these processes and uncover novel therapeutic approaches. The objective of this study is to enhance future medical therapies by clarifying prospective targets for treatment and the mechanisms to prevent damage to the pancreas.

## Materials and Methods

### Ethics Approval

The study was conducted according to the ethical standards specified in the 1964 Declaration of Helsinki. The ethical rules for research and publication were followed in our study. Our study was approved by the ethics committee of Recep Tayyip Erdoğan University Animal Experiments Ethical Committee (Rize, Türkiye, approval number: 2023/09; approval date: 14.02.2023).

The 36 male Sprague-Dawley rats (3–4 months) used in this study were obtained from the Animal Research Unit, Faculty of Medicine, Recep Tayyip Erdoğan University. The rats were cared for under optimal laboratory conditions, which involved 20–26 °C, 50–70% humidity, and a 12:12-h light-darkness cycle according to the principles in the National Research Council’s Guide for the Care and Use of Laboratory Animals. The animals were housed in 36 × 23 × 21 cm^3^ polypropylene cages with 6 rats in each group. The subjects were also allowed ad libitum access to standard chow and water. After the study was completed, all animals were euthanized under high-dose anesthesia. All stages of the study were carried out at the Recep Tayyip Erdoğan University Experimental Animals Unit. ARRIVE (animal research: reporting in vivo experiments) guidelines were followed at all stages of the study [24].

### Experimental Animals (Fig. 1)

**Fig. 1:**
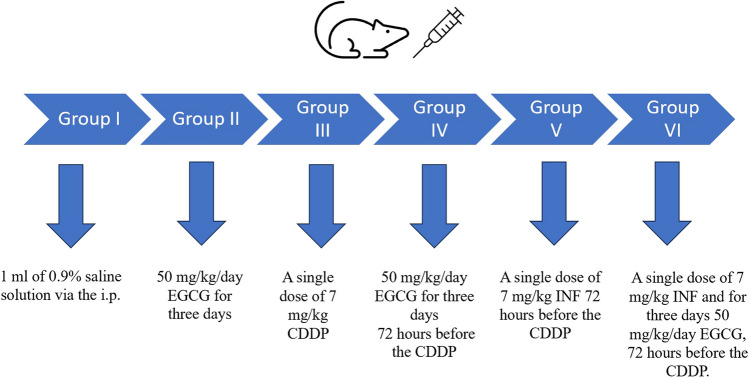
Experimental study design

Sprague-Dawley rats (male, *n* = 36, weight = 320 ± 42 g) aged 3–4 months were randomized into six equal groups. The sample size in the study was calculated according to the formulas given below:

$$n\:=\:\mathrm{DF}/k\:+\:1$$

$$\mathrm{Minimum}\;n\:=\:10/k\:+\:1$$

$$\mathrm{Maximum}\;n\:=\:20/k\:+\:1$$

$$\mathrm{Minimum}\;N\:=\:\mathrm{minimum}\;n\;\times\;k$$

$$\mathrm{Maximum}\;N\:=\:\mathrm{maximum}\;n\;\times\;k$$

where *N* = total number of subjects, *k* = number of groups, and *n* = number of subjects per group. According to one-way ANOVA, based on the acceptable range of degrees of freedom (DF), the DF in the formulas was replaced by minimum (10) and maximum (20) DFs to obtain the minimum and maximum number of animals per group [25–27]. Thirty-six rats were divided into six groups. The experimental design of the study is shown in Table 1. The rats in group 1 (*n* = 6; control) received only 1 mL of 0.9% saline solution via the intraperitoneal route (i.p.). Group 2 (*n* = 6; EGCG) received 50 mg/kg/day EGCG via the i.p. route for 3 days [28]. Rats in group 3 (*n* = 6; CDDP) were given a single dose of 7 mg/kg cisplatin via the i.p. route. Rats in group 4 (*n* = 6, CDDP + EGCG) were administered 50 mg/kg/day EGCG for 3 days, starting 72 h before cisplatin administration [28]. Rats in group 5 (*n* = 6, CDDP + INF) received a single i.p. dose of 7 mg/kg infliximab (INF) 72 h before the application of 7 mg/kg cisplatin. Infliximab reaches a steady blood concentration after the third day. Therefore, we administered infliximab 3 days before CDDP chemotherapy [6]. Finally, rats in group 6 (*n* = 6, CDDP + EGCG + INF) were administered a single dose of 7 mg/kg INF and 50 mg/kg/day EGCG for 3 consecutive days, starting 72 h before a single intraperitoneal administration of 7 mg/kg CDDP [28, 29]. The doses of infliximab, EGCG, and cisplatin were determined based on previous studies in the literature [28–32]. On the fourth day, after the completion of all procedures, all rats were killed by decapitation after inducing appropriate hibernation with 100 mg/kg ketamine HCl, 10 mg/kg xylazine, and 0.5 mL/kg fentanyl citrate intraperitoneally [33]. All of the pancreatic tissue was removed from the rats and divided into two equal parts in the coronal plane. One part was saved for biochemical analysis, and the other for histopathological and immunohistochemical analysis.

**Table 1:** Experimental study design

Groups	96 hours	72 hours	48 hours	24 hours	0 hours
Group 1	1 mL 0.09% NaCl	1 mL 0.09% NaCl	1 mL 0.09% NaCl	–	Sacrification
Group 2	50 mg/kg EGCG	50 mg/kg EGCG	50 mg/kg EGCG	–	Sacrification
Group 3	–	–	–	7 mg/kg CDDP	Sacrification
Group 4	50 mg/kg EGCG	50 mg/kg EGCG	50 mg/kg EGCG	7 mg/kg CDDP	Sacrification
Group 5	7 mg/kg INF	–	–	7 mg/kg CDDP	Sacrification
Group 6	50 mg/kg EGCG + 7 mg/kg INF	50 mg/kg EGCG	50 mg/kg EGCG	7 mg/kg CDDP	Sacrification

*NaCl* sodium chloride; *EGCG* epigallocatechin-3-gallate; *CDDP* cisplatin, cis-diamminedichloroplatinum; *INF* infliximab

### Chemicals

Anesthesia was administered using ketamine hydrochloride (Ketalar, 100 mg/kg, Pfizer İlaçları Ltd., İstanbul, Türkiye) and xylazine hydrochloride (Rompun, 10 mg/kg, Bayer, USA). Analgesia was administered using fentanyl citrate (Talinat, 0.5 mg/10 ml, Vem Pharmaceutical Industry Inc., Ankara, Türkiye). Cisplatin DBL 1 mg/mL was obtained from Orna İlac Tekstil Kimyevi Mad. San. ve Dıs. Tic. Ltd. Sti. (Beykoz/İstanbul, Türkiye), while infliximab (Remicade 100 mg/vial) was obtained from Merck Sharp Dohme Pharma Ltd. Epigallocatechin 3-gallate ((-)-epigallocatechin gallate, E4143-50MG) and all chemicals used in laboratory experiments were provided by Sigma-Aldrich Chemical Co. and Merck (Germany).

### Biochemical Analysis

Pancreatic tissue specimens weighing 0.1 g excised from the rats were treated with 20 mM sodium phosphate + 140 mM potassium chloride and 1 L homogenization buffer (pH 7.4). No other solution was used for the homogenate except for sodium phosphate and potassium chloride homogenization buffer. It was homogenized with a homogenizer (QIAGEN TissueLyser) for 30 Hz/5 min. In the next stage, using the supernatant obtained from centrifugation at 800 g for 10 min at 4 °C, total thiol (TT) group and thiobarbituric acid reactive substance (TBARS) assays were performed. The TBARS assay followed the study by Ohkawa et al. [34]. A mixture of 200 µL tissue supernatant, 50 µL of 8.1% SDS (sodium dodecyl sulfate), 375 µL of 20% acetic acid (v/v) (pH 3.5), and 375 µL of 0.8% thiobarbituric acid (TBA) was vortexed, and the reaction was left to incubate in a boiling water bath for 1 h. Following incubation, it was cooled in ice water for 5 min and centrifuged at 750 g for 10 min. The resulting pink color was assessed by a spectrophotometer at 532 nm. The results were expressed as nanomoles per gram (nmol/g) of tissue.

The determination of total thiol groups followed the spectrophotometric method of Sedlak and Lindsay [35]. The Ellman reagent was used to determine the –SH groups. Briefly, first of all, 50 µL of supernatant, 200 µL of 3 M Na2HPO4, and 50 µL of Ellman’s reagent were added. The yellow color of the free sulfhydryl groups in the tissue homogenate was formed with Ellman’s reagent and read at 412 nm in the spectrophotometer. The results were expressed as micromoles per gram (µmol/g) of tissue.

### Histopathological Analysis

Standard histological techniques were used to examine the pancreatic tissue of rats. Rat pancreatic tissue samples were originally measured using checkered paper and then preserved in a formalin solution for histological investigation. The tissues were dehydrated using a series of ethanol solutions of increasing concentrations, followed by a cleaning step in xylol. Finally, the tissues were embedded in paraffin. Ultimately, thin slices were sliced, placed on slides, and treated with Harris Hematoxylin and Eosin G for microscopic examination.

### Immunohistochemical Analysis

The immunohistochemical analysis of rat pancreatic tissue specimens used an insulin primary antibody kit (ab181547, 1/250, Abcam Inc., Cambridge, UK), a glucagon primary antibody kit (ab92517, 1/200, Abcam Inc., Cambridge, UK), TNF-α primary antibody kit (ab307164, 1/200, Abcam Inc., Cambridge, UK), and the 8-hydroxy-2′-deoxyguanosine kit (8-OHdG, 1/200, ab62623, Cambridge, UK). In the IHC analysis, pancreatic tissue specimens were incubated with the primary and secondary antibodies (Goat Anti-Rabbit IgG H&L, HRP, ab97051, Abcam Inc., Cambridge, UK) for 1 h using a BOND-MAX ICH/ISH device (Leica Biosystems, Australia). Dilution rates of primary and secondary antibodies were applied, as shown in Table 2. Diaminobenzidine chromogen solution (DAB Substrate Kit, ab64238, Abcam, UK) was applied to the pancreatic tissue specimens incubated with the primary and secondary antibodies for visualization under the light microscope. The tissues were counterstained with Harris Hematoxylin (Merck KGaA, Darmstadt, Germany) and mounted with an appropriate mounting solution.

**Table 2:** Information on primary and secondary antibodies used in immunohistochemical analysis

Primary antibody	Dilution rate	Positive control (according to manufacturer’s recommendation)	Secondary antibody	Dilution rate
Primary insulin antibody (ab181547, Abcam Inc., Cambridge, UK)	1/250 (diluted with antibody diluent (ab64211, Abcam Inc., Cambridge, UK)	Pancreas tissue	Goat Anti-Rabbit IgG H&L (HRP, ab97051, Abcam Inc., Cambridge, UK)	1/200 (diluted with antibody diluent (ab64211, Abcam Inc., Cambridge, UK)
Primary glucagon antibody (ab92517, Abcam Inc., Cambridge, UK)	1/200 (diluted with antibody diluent (ab64211, Abcam Inc., Cambridge, UK)	Pancreas tissue	Goat Anti-Rabbit IgG H&L (HRP, ab97051, Abcam Inc., Cambridge, UK)	1/200 (diluted with antibody diluent (ab64211, Abcam Inc., Cambridge, UK)
Primary TNF-α antibody (ab307164, Abcam Inc., Cambridge, UK)	1/200 (diluted with antibody diluent (ab64211, Abcam Inc., Cambridge, UK)	Spleen	Goat Anti-Rabbit IgG H&L (HRP, ab97051, Abcam Inc., Cambridge, UK)	1/200 (diluted with antibody diluent (ab64211, Abcam Inc., Cambridge, UK)
Primary 8-OHdG antibody (ab62623, Abcam Inc., Cambridge, UK)	1/200 (diluted with antibody diluent (ab64211, Abcam Inc., Cambridge, UK)	Rat back skin	Goat Anti-Rabbit IgG H&L (HRP, ab97051, Abcam Inc., Cambridge, UK)	1/200 (diluted with antibody diluent (ab64211, Abcam Inc., Cambridge, UK)

#### The Terminal Deoxynucleotidyl Transferase dUTP Nick End Labeling (TUNEL) Method

In situ apoptosis detection kit (TUNEL Assay Kit- HRP-DAB, ab206386, Abcam Inc., Cambridge, UK) was used in the TUNEL method. Sections of pancreatic tissue were embedded in paraffin and dehydrated. In the next step, the TUNEL method was applied according to the manufacturer’s guidelines and incubated with DAB chromogen (DAB Substrate Kit, ab64238, Abcam, UK). According to the manufacturer’s recommendation, spleen tissue was selected as a positive control. The organs utilized as positive and negative controls in the investigation were acquired from rats belonging to the control group. Twenty-five randomly selected fields per rat pancreatic tissue section were scored for TUNEL positivity by an experienced histopathologist blind to the study groups (under ×40 magnification objectives).

### Semi-quantitative Analysis

In the histopathological analysis of rat pancreatic tissues, a histopathological damage score (HDS) was calculated in accordance with the earlier studies on pancreatic tissue toxicity about findings of necrosis in islets of Langerhans cells, necrosis (accompanied by diffuse loss of cytoplasm) in exocrine acinar epithelial cells (acinar necrosis, fat necrosis) and edema as shown in Table 3 [8, 36]. Twenty-five different randomly selected fields per rat pancreatic tissue section were scored by an experienced histopathologist blind to the study groups.

**Table 3:** Pancreas histopathological damage score (HDS)

Score	Findings
Edema	
0	None
1	Interlobular septae (local expansion)
2	Interlobular septae (diffuse expansion)
3	Interacinar septae (focal + diffuse expansion)
Acinar necrosis	
0	None
1	Focal or/and diffuse occurrence of 1–4 necrotic cells
2	Diffuse occurrence of 5–10 necrotic cells
3	Diffuse occurrence of 11–16 necrotic cells
Fat necrosis	
0	None
1	2 foci
2	4 foci
3	6 foci

Cells showing immune positivity in rat pancreatic tissue sections incubated with the primary and secondary antibodies using indirect immunohistochemical methods were scored as presented in Table 4 [37]. An experienced histopathologist who was blind to the study groups scored thirty different randomly selected fields per rat pancreatic tissue section.

**Table 4:** Semi-quantitative analysis

Score	Results
0	Less than 5%
1	Between 5–25%
2	Between 25–50%
3	Between 51–75%
4	More than 75%

In our study, the distribution of endocrine cells showing insulin and glucagon positivity in the islets of Langerhans was scored in accordance with the studies of El Agaty and Ibrahim Ahmed and O’Brein et al. [38, 39]. In each pancreatic tissue preparation, the nuclei of all islet cells were counted in 25 different areas using ×40 magnification. By counting the islets, the number of α (cells showing glucagon positivity) and β-cells (cells showing insulin positivity) (α*n* and β*n*) and the sum of islet cell nuclei (the number of glucagon-positive and insulin-positive cells in the islets of Langerhans and the number of immune negative whose nuclei are stained blue with Harris Hematoxylin) were determined and calculated according to the following equation:

$$\begin{array}{cc}\mathrm{Alfa}-\mathrm p=\alpha n/\mathrm{In}\;\times100,&\mathrm{Beta}-\mathrm p=\end{array}\beta n/\mathrm{In}\;\times100$$

### Planned Statistical Analyses (Table 5)

**Table 5:** Summary of outcomes and planned statistical analyses

Outcomes	Analyses	Planned statistical analyses
Primary outcomes	Pancreas histopathological damage score (HDS)	One-way ANOVA or non-parametric test
Secondary outcomes	Edema score	
Secondary outcomes	Acinar necrosis score	
Secondary outcomes	Fat necrosis score	
Primary outcomes	Biochemical analysis	One-way ANOVA or non-parametric test
Secondary outcomes	MDA levels	
Secondary outcomes	GSH levels	
Primary outcomes	İmmunohistochemical positivity score	One-way ANOVA or non-parametric test
Secondary outcomes	Insulin positivity score	
Secondary outcomes	Glucagon positivity score	
Secondary outcomes	Apoptosis score (TUNEL methods)	
Secondary outcomes	TNF-α positivity score	
Secondary outcomes	8-OHdG positivity score	

The data acquired from histopathological, immunohistochemical, and biochemical analyses were assessed for normal distribution using Shapiro-Wilk's test, Q-Q plot, Skewness-Kurtosis values, and Levene’s tests using the SPSS 20.0 (IBM Corp., Armonk, NJ, USA) statistics software. Quantitative (biochemical) parametric data obtained from the analyses were expressed as mean ± standard error values. Non-parametric data obtained from the semi-quantitative analyses (histopathological and immunohistochemical analyses) were expressed as median, 25%, and 75% interquartile range values. For quantitative (biochemical) parametric data, differences between the groups were analyzed using one-way ANOVA and Tukey’s HSD tests. On the other hand, non-parametric data were analyzed using the Kruskal-Wallis test, followed by the Mann-Whitney *U* test with Bonferroni's correction. In determining the differences between groups of parametric and non-parametric data, a *p*-value < 0.05 was taken as statistically significant.

### Measures to Prevent Bias

Animals were randomized between groups to prevent allocation bias (as per Sect. 2.1.2., ‘Stratified Allocation’) [25]. To avoid analytical bias, all samples retained for analysis were recorded using a random number generator (https://www.graphpad.com/quickcalcs/randomN2/). A third party carried out this and kept the random number key secret from or withheld it from the researchers running primary sample analyses. A third party recording and hiding the experimental group’s identity (A, B, or C) by coming up with alternate codenames for each did the decoding and reassembly of the data.

## Results

### Biochemical Analysis

#### Malondialdehyde (MDA) Levels (TBARS Assay)

There was a statistical difference between the groups in the One-Way ANOVA test for MDA levels (df = 5, F = 9.323). We determined higher pancreatic MDA levels in the CDDP group compared to the EGCG groups (Table 6, *p* = 0.001). In contrast, we determined lower pancreatic MDA levels in the CDDP + EGCG and CDDP + INF groups compared to the cisplatin group (Table 6, *p* = 0.001). Similarly, we observed lower pancreatic MDA levels in the CDDP + EGCG + INF group compared to the CDDP group (Table 6, *p* = 0.001). MDA levels in the CDDP + EGCG and CDDP + INF groups were similar, and no statistical difference was detected (Tables 1 and 61.83 ± 0.22, 11.9 ± 0.20, respectively, *p* = 0.999).

**Table 6:** Biochemical analysis score results (mean ± 1(standard error))

Group	MDA (nmol/g tissue)	GSH (µmol/g tissue)
Control	12.26 ± 0.58	5.20 ± 0.09
EGCG	12.11 ± 0.60	5.00 ± 0.07
Cisplatin (CDDP)	14.93 ± 0.23^a,b^	2.62 ± 0.07^a,b^
CDDP + EGCG	11.83 ± 0.22^c^	4.88 ± 0.08^c^
CDDP + INF	11.90 ± 0.20^c^	5.05 ± 0.09^c^
CDDP + EGCG + INF	12.22 ± 0.23^c^	4.89 ± 0.08^c^

One-way ANOVA-Tukey HSD

^a^*p*=0.001: compared to the control group

^b^*p*=0.001: compared to the EGCG group

^c^*p*=0.001: compared to the CDDP group

#### Total Thiol (TT) Levels

There was a statistical difference between the groups in the one-way ANOVA test for GSH levels (df = 5, *F* = 146.628). We observed lower pancreatic GSH levels in the CDDP group compared to the EGCG group (Table 6, *p* = 0.001). In contrast, we determined higher pancreatic GSH levels in the CDDP + EGCG and CDDP + INF groups compared to the CDDP group (Table 6, *p* = 0.001). Similarly, we observed higher pancreatic GSH levels in the CDDP + EGCG + INF group compared to the CDDP group (Table 6, *p* = 0.001). GSH levels in the CDDP + EGCG and CDDP + INF groups were similar, and no statistical difference was detected (Table 6, 4.88 ± 0.08, 5.05 ± 0.09, respectively, *p* = 0.0.662).

### Histopathological Analysis

**Fig. 2:**
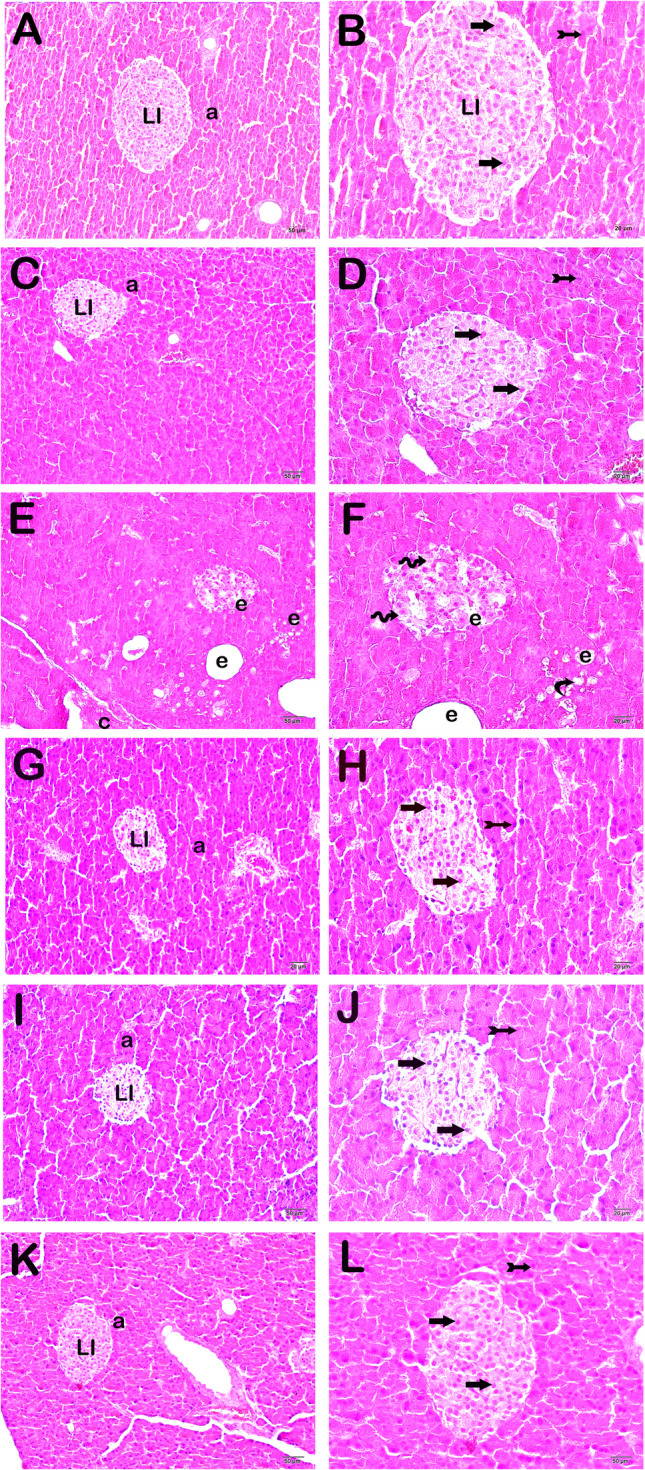
Representative light microscopic image of H&E-stained pancreatic tissue.  Langerhans islet (LI), acinus (a).  **A **(×20), **B **(×40) control group: islets of Langerhans (LI) and exocrine acini of normal structure. **C **(×20), **D **(×40) EGCG group: islets of Langerhans (LI) and exocrine acini of typical structure. ** E **(×20), **F **(×40) CDDP group: endocrine cells (spiral arrow) in the islets of Langerhans (LI) and exocrine acini showing necrosis (curved arrow). Fat necrosis accompanied by extensive edematous areas (e) can also be seen. **G **(×20), **H **(×40) CDDP + EGCG group: fewer necrotic cells were found in the endocrine cells (arrow) in the islets of Langerhans (LI) and exocrine acini (tailed arrow). A decrease in fat necrosis and the accompanying extensive edematous areas (e) can also be seen. **I **(×20), **J **(×40) CDDP + INF group: a decrease can be seen in the islets of Langerhans (LI) and exocrine acini, as well as fat necrosis and the accompanying extensive edematous areas. Diffuse typical endocrine cells (arrow) and acinar cells (tailed arrow). **K **(×20), **L **(×40) CDDP + EGCG + INF group: a decrease in necrotic endocrine cells in the islets of Langerhans (LI) and in epithelial cells in exocrine acini can be seen. A decrease in fat necrosis and the accompanying extensive edematous areas can also be seen. Additionally, diffuse typical endocrine cells (arrow) and exocrine acinar epithelial cells (tailed arrow) of typical structure can be seen

Pancreas histopathological damage score (HDS) was calculated by considering necrotic cells, fat necrosis, and edematous areas in accordance with the studies on cisplatin-induced pancreatic damage. There was a statistical difference between the groups in the one-way ANOVA test for HDS (df = 5, *F* = 142.094). We observed normal islets of Langerhans and exocrine acini in the pancreatic tissues of the control group (Fig. 2A, B; Table 7, HDS 0.5 (0–1)). Similarly, we observed that endocrine cells in the islets of Langerhans and epithelial cells in exocrine acini were of normal structure in the EGCG group (Fig. 2C, D; Table 7, HDS 1 (0–1)). In contrast, rat pancreatic sections of the CDDP group presented diffuse necrotic endocrine cells and necrotic epithelial cells in exocrine acini. In addition, we observed extensive fat necrosis and edematous areas (Fig. 2E, F; Table 7, *p* = 0.001, HDS 7 (6–8)). In pancreatic tissue sections of the CDDP + EGCG group, we determined reductions in the fat necrosis and the extensive edematous areas in the islets of Langerhans and exocrine acini (Fig. 2G, H; Table 7, *p* = 0.001, HDS 2 (1–3)). Similarly, the CDDP + INF group showed reduced fat necrosis and less extensive edematous areas in the islets of Langerhans and exocrine acini (Fig. 2I, J; Table 7, *p* = 0.001, HDS 2 (1–2)). In the CDDP + EGCG + INF group, we found typical endocrine cells in the islets of Langerhans and epithelial cells in exocrine acini. In addition, we observed reduced fat necrosis and less extensive edematous areas (Fig. 2K, L; Table 7, *p* = 0.001, HDS 1 (0.5–1)).

**Table 7:** Pancreas histopathological damage score (HDS) results (median (25–75% interquartile range))

Group	Acinar necrosis	Fat necrosis	Edema	HDS
Control	0 (0–0)	0 (0–0)	0 (0–0)	0.5 (0–1)
EGCG	0 (0–0.5)	0 (0-0.5)	0 (0–0)^a^	1 (0–1)
Cisplatin (CDDP)	2 (2–3)^a,b^	2 (2–3)^a,b^	2 (2–3)^a,b^	7 (6–8)^a,b^
CDDP + EGCG	1 (0–1)^c^	0 (0–1)^c^	0.5 (0–1)^c^	2 (1–3)^c^
CDDP + INF	0.5 (0–1)^c^	1 (0–1)^c^	0 (0–1)^c^	2 (1–2)^c^
CDDP + EGCG + INF	0 (0–1)^c^	0 (0–1)^c^	0 (0–1)^c^	1 (0.5-1)^c,d,e,f^

The Mann-Whitney *U* test with Bonferroni's corrections

^***a***^*p*=0.001: compared to the control group, ^***b***^*p*=0.001: compared to the EGCG group, ^***c***^*p*=0.001: compared to the CDDP group, ^***d***^*p*=0.018: compared to the control group, ^***e***^*p*=0.045: compared to the EGCG group, ^***f***^*p*=0.002: compared to the CDDP + INF group

### Immunohistochemical Analysis

#### Insulin Positivity

**Fig. 3:**
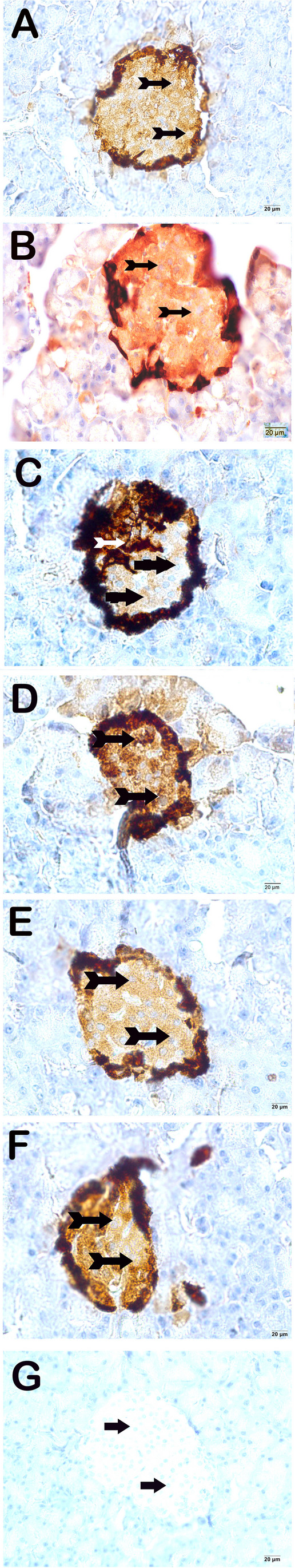
Representative light microscopic image of pancreatic tissue cells incubated with insulin primary antibody.  **A **(×20) control group: β-cells (tailed arrow) showing extensive insulin positivity in normal islets of Langerhans. **B **(×20) EGCG group: typical β-cells (tailed arrow) showing extensive insulin positivity. **C **(×20) CDDP group: lower insulin positivity in the islets of Langerhans with necrotic cells (arrow). **D **(×20) CDDP + EGCG group: higher insulin positivity in endocrine cells (tailed arrow) in the islets of Langerhans. **E **(×20) CDDP + INF group: extensive insulin positivity in endocrine cells (tailed arrow) in the islets of Langerhans. **F **(×20) CDDP + EGCG + INF group: extensive insulin positivity in the cells (tailed arrow) in the islets of Langerhans. **G **(×20) negative control: a representative light microscopic screen image of pancreatic tissue stained with Harris Hematoxylin alone shows that endocrine cells (arrow) are immuno-negative

There was a statistical difference between the groups in the one-way ANOVA test for insulin positivity (df = 5, *F* = 33.416). Sections of pancreatic tissue incubated with insulin primary antibody showed extensive insulin positivity in β-cells in normal islets of Langerhans in the control group (Fig. 3A; Table 8, *p* = 0.001, insulin positivity score 2.5 (3–2)). In the EGCG group, we observed numerous β-cells in the islets of Langerhans that showed extensive insulin positivity (Fig. 3B; Table 8, *p* = 0.001, insulin positivity score 2 (2–3)). In contrast, in the CDDP group, we observed fewer β-cells in the islets of Langerhans that were positive for insulin compared to the control group (Fig. 3C; Table 8, *p* = 0.001, insulin positivity score 0 (0–1)). In the CDDP + EGCG and CDDP + INF groups, we found an increase in β-cells showing extensive insulin positivity compared to the CDDP group (Fig. 3D, E; Table 8, *p* = 0.001, insulin positivity score: 2 (2–2.5) and 2 (2–3), respectively). Similarly, in the CDDP + EGCG + INF group, we determined an increase in β-cells in the islets of Langerhans that showed extensive insulin positivity compared to the CDDP group (Fig. 3F; Table 8, *p* = 0.001, insulin positivity score 3 (2–3)).

**Table 8:** Immunohistochemical analysis (median 25–75% interquartile range))

Group	Insulin positivity score	Glucagon positivity score	TUNEL positivity score	TNF-α positivity score	8-OHdG positivity score
Control	2.5 (2–3)	2 (2–2)	0 (0–0)	0 (0–0)	0 (0–0)
EGCG	2 (2–3)^a^	2 (2–2)^a^	0 (0–0.5)^a^	0 (0–0.5)^a^	0 (0–1)^a^
Cisplatin (CDDP)	0 (0–1)^a,b^	0 (0–1)^a,b^	2 (2–2.5)^a,b^	2 (2–3)^a,b^	
CDDP + EGCG	2 (2-2.5)^c^	2 (2–3)^c^	0 (0–0)^c^	0 (0–0)^c^	0 (0–1)^c^
CDDP + INF	2 (2–3)^c^	2 (2–2.5)^c^	0 (0–1)^c^	0 (0–0)^c^	0 (0–1)^c^
CDDP + EGCG + INF	3 (2–3)^c^	2 (2–2)^c^	0 (0–0)^c^	0 (0–0)^c^	0 (0–1)^c^

The Mann-Whitney *U* test with Bonferroni's corrections

^***a***^*p*=0.001: compared to the control group, ^***b***^*p*=0.001: compared to the EGCG group, ^***c***^*p*=0.001: compared to the CDDP group

**Fig. 4:**
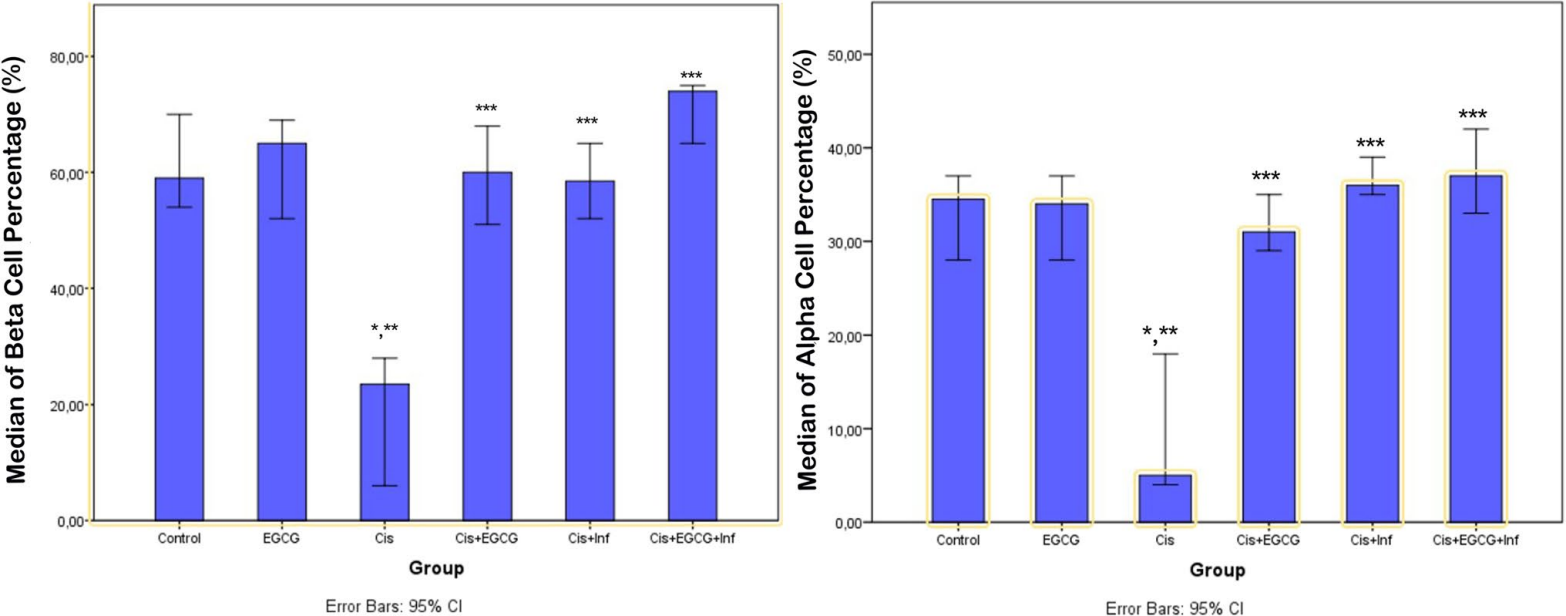
Percentage area (%) of anti-insulin and anti-glucagon antibody reaction in islets (according to El Agaty and Ibrahim Ahmed and O’Brien et al. [38, 39]).  *p  < 0.001: compared to the control group, **p  < 0.001: compared to the EGCG group, ***p  < 0.001: compared to the CDDP group

According to studies conducted by El Agaty and Ibrahim Ahmed and O’Brien et al. [38, 39], the percentage distribution of beta cells showing insulin positivity in the islets of Langerhans was evaluated. The results showed that the control group had 59% (54–70.5) of beta cells showing insulin positivity, while the EGCG group had 65% (52–70). These values were statistically similar (Figs. 3A, B and 4). Contrarily, the distribution of insulin-positive beta cells in the CDDP group decreased significantly to 21% (5–28) compared to the control group (Figs. 3C and 4, *p* < 0.001). However, we observed that the distribution of insulin-positive beta cells increased to 60% (50.5–68.5) and 58.5% (71.5–65) in the CDDP + EGCG and CDDP + INF groups, respectively, compared to the CDDP group (Figs. 3C–E and 4, *p* < 0.001 and *p* < 0.001, respectively). Similarly, an increase in insulin-positive beta cells to 74% (65–75.5) was observed in the CDDP + EGCG + INF group, as compared to the CDDP group (Figs. 3F and 4, *p* < 0.001).

#### Glucagon Positivity

**Fig. 5:**
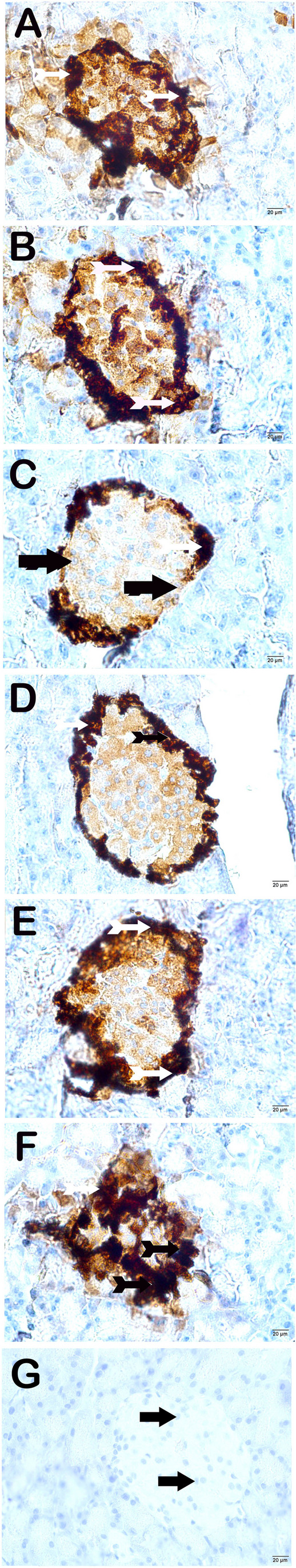
Representative light microscopic image of pancreatic tissue cells incubated with glucagon primary antibody.  **A **(×20) control group: normal α-cells (tailed arrow) showing extensive glucagon positivity in the periphery of the islets of Langerhans. **B **(×20) EGCG group: typical α-cells (tailed arrow) showing extensive glucagon positivity. **C **(×20) CDDP group: lower glucagon positivity in α-cells (arrow) in the islets of Langerhans. **D **(×20) CDDP + EGCG group: higher glucagon positivity in α-cells (tailed arrow) in the periphery of the islets of Langerhans. **E **(×20) CDDP + INF group: extensive glucagon positivity in α-cells (tailed arrow). **F **(×20) CDDP + EGCG + INF group: extensive glucagon positivity in endocrine cells (tailed arrow) in the periphery of the islets of Langerhans. **G **(×20) negative control: a representative light microscopic screen image of pancreatic tissue stained with Harris Hematoxylin alone shows that endocrine cells (arrow) are immuno-negative

There was a statistical difference between the groups in the one-way ANOVA test for glucagon positivity (df = 5, *F* = 44.902). Upon the incubation of sections of pancreatic tissue with glucagon primary antibody, we determined numerous α-cells showing extensive immune positivity in the control and EGCG groups (Fig. 5A, B; Table 8, *p* = 0.001, glucagon positivity scores, respectively, 2 (2–2) and 2 (2–2)). In contrast, in the CDDP group, we observed fewer glucagon-positive α-cells (Fig. 5C; Table 8, *p* = 0.001, glucagon positivity score 0 (0–1)). On the other hand, we observed an increase in the number of α-cells showing extensive glucagon positivity in the CDDP + EGCG and CDDP + INF groups (Fig. 5D, E; Table 8, *p* = 0.001, glucagon positivity scores 2 (2–3) and 2 (2–2.5), respectively). Similarly, in the CDDP + EGCG + INF combination treatment group, we observed an increase in the number of α-cells showing extensive glucagon positivity (Fig. 5F; Table 8, *p* = 0.001, glucagon positivity score 2 (2–2)).

When analyzing the percentage distribution of alpha cells that exhibit glucagon positivity in the islets of Langerhans, we found that the control group had a rate of 74.5% (28–37), while the EGCG group was recorded at 34% (27.5–37.5), which was statistically similar as shown in Figs. 4 and 5A, B. In contrast, the CDDP group had a significant decrease of alpha cells showing glucagon positivity at 5% (3.5–18.5) compared to the control group (Figs. 4 and 5C, *p* < 0.001). However, we observed that the alpha cells that showed glucagon positivity increased to 31% (28.5–35) and 36% (73.5–40.5) in the CDDP + EGCG and CDDP + INF groups, respectively, compared to the CDDP group (Figs. 4 and 5C–E, *p* < 0.001 and *p* < 0.001, respectively). Furthermore, the CDDP + EGCG + INF group had an even higher increase of alpha cells showing glucagon positivity at 37% (32.5–43.5) compared to the CDDP group (Figs. 4 and 5F, *p* < 0.001).

#### TUNEL Positivity (Apoptosis Score)

**Fig. 6:**
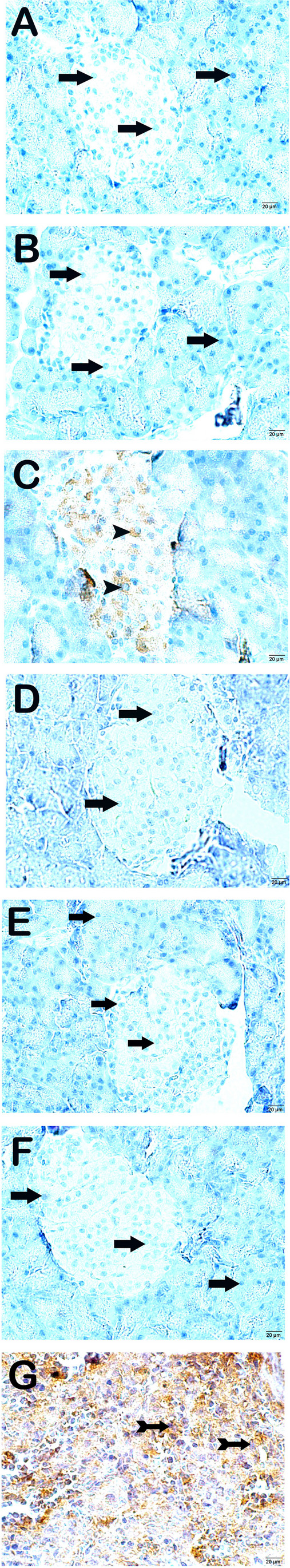
Representative light microscopic image of apoptotic cells in pancreatic tissue labeled with the TUNEL method.  **A **(×20) control group: normal immune-negative endocrine cells (arrow) in the islets of Langerhans. **B **(×20) EGCG group: typical immune-negative endocrine cells (arrow) in the islets of Langerhans. **C **(×20) CDDP group: numerous apoptotic endocrine cells found in the islets of Langerhans (arrowhead). **D **(×20) CDDP + EGCG group: fewer apoptotic cells (arrow) in the islets of Langerhans. **E **(×20) CDDP + INF group: fewer immune-positive cells in the islets of Langerhans. **F **(×20) CDDP + EGCG + INF group: typical immune-negative endocrine cells found in the islets of Langerhans. **G** (×20) Positive control: immune-positive cells are observed in the spleen tissue (tailed arrow)

There was a statistical difference between the groups in the one-way ANOVA test for TUNEL positivity (df = 5, *F* = 36.435). On examination of the sections of pancreatic tissue subjected to the TUNEL method in order to identify apoptotic cells under a light microscope, we observed normal histologic structure of pancreatic and exocrine acinar epithelial cells in the control and EGCG groups (Fig. 6A, B; Table 8, *p* = 0.001, apoptosis scores 0 (0–0) and 0 (0–0.5), respectively). However, we determined an increase in endocrine cells showing extensive immune positivity in the CDDP group (Fig. 6C; Table 8, *p* = 0.001, apoptosis score 2 (2–2.5)). In the CDDP + EGCG, CDDP + INF, and CDDP + EGCG + INF groups, we observed a decrease in apoptotic cells showing TUNEL positivity, primarily in endocrine cells in the islets of Langerhans (Fig. 6D–F; Table 8, *p* = 0.001, apoptosis scores 0 (0–0), 0 (0–1), and 0 (0–0), respectively).

#### TNF-α (Tumor Necrosis Factor-α) Positivity

**Fig. 7:**
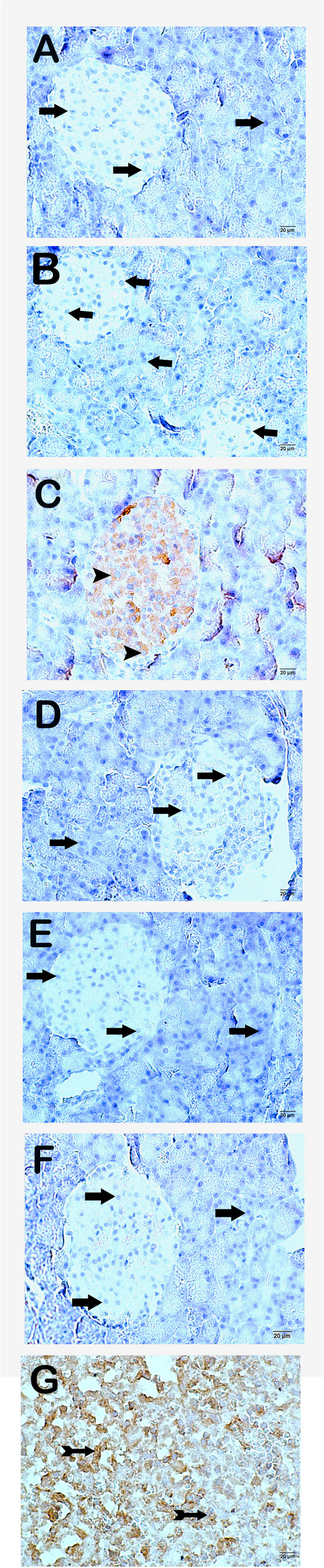
Representative light microscopic image of pancreatic tissue sections incubated with the TNF-α primary antibody.  **A **(×20) Control group: normal TNF-α-negative endocrine cells (arrow) in the islets of Langerhans. **B **(×20) EGCG group: typical endocrine cells (arrow) in the islets of Langerhans that are TNF-α-negative. **C **(×20) CDDP group: extensive TNF-α positivity in numerous endocrine cells (arrowhead) found in the islets of Langerhans. **D **(×20) CDDP + EGCG group: fewer endocrine cells with extensive TNF-α positivity in the islets of Langerhans (arrow). **E **(×20) CDDP + INF group: reduced TNF-α positivity in the islets of Langerhans (arrow). **F **(×20) CDDP + EGCG + INF group: typical immune-negative endocrine cells found in the islets of Langerhans (arrow). **G** (×20) Positive control: immune-positive cells (tailed arrow) are observed in the spleen tissue

There was a statistical difference between the groups in the one-way ANOVA test for TNF-α positivity (df = 5, *F* = 81.473). Sections of pancreatic tissue from the control and EGCG groups that were incubated with the TNF-α primary antibody showed typical immune cells that were immune-negative for the TNF-α primary antibody (Fig. 7A, B; Table 8, *p* = 0.001, TNF-α positivity scores 0 (0–0) and 0 (0–0.5)). In the CDDP group, we determined an increase in TNF-α-positive endocrine cells in the islets of Langerhans (Fig. 7C; Table 8, *p* = 0.001, TNF-α positivity score 2 (2–3)). In contrast, in pancreatic tissue sections of the CDDP + EGCG, CDDP + INF, and CDDP + EGCG + INF groups, we observed fewer TNF-α-positive endocrine cells in the islets of Langerhans (Fig. 7D–F; Table 8, *p* = 0.001, TNF-α positivity scores 0 (0–0), 0 (0–0), and 0 (0–0), respectively).

#### 8-OHdG (8-Hydroxy-2-Deoxyguanosine) Positivity

**Fig. 8:**
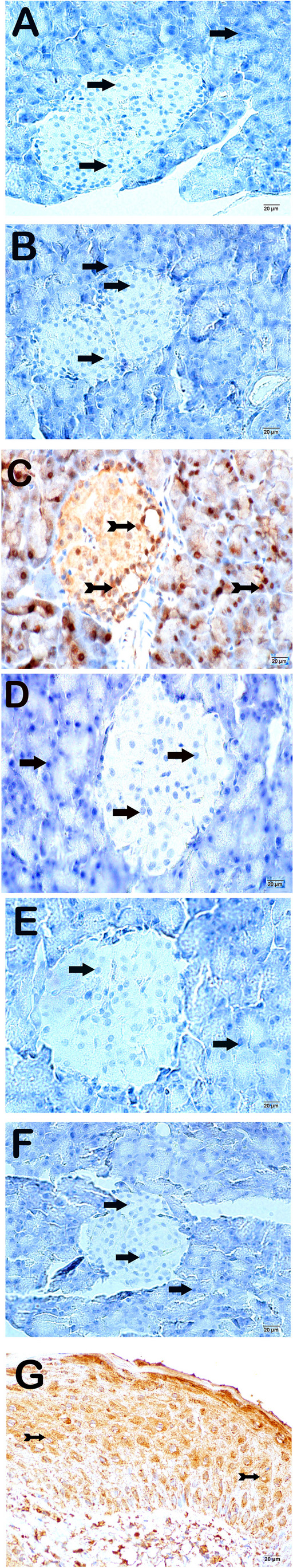
Representative light microscopic image of pancreatic tissue sections incubated with the 8-OHdG primary antibody.  **A **(×20) Control group: normal islets of Langerhans and exocrine acini that are immune-negative. **B **(×20) EGCG group: typical endocrine cells (arrow) in the islets of Langerhans and typical exocrine acinar epithelial cells (arrow) that are 8-OHdG-negative. **C **(×20) CDDP group: extensive 8-OHdG positivity in numerous endocrine cells found in the islets of Langerhans (tailed arrow). In addition, the exocrine pancreatic epithelium (tailed arrow) with extensive immune positivity can be seen. **D **(×20) CDDP + EGCG group: lower 8-OHdG positivity in endocrine cells in the islets of Langerhans and epithelial cells in exocrine acini (arrow). **E **(×20) CDDP + INF group: fewer immune-positive cells in the islets of Langerhans and exocrine acini (arrow). **F **(×20) CDDP + EGCG + INF group: typical endocrine cells in the islets of Langerhans and epithelial cells in the exocrine pancreas (arrow). In addition, the cells, primarily endocrine cells in the islets of Langerhans, are immune-negative (arrow). **G** (×20) Positive control: immune-positive cells (tailed arrow) are observed in sections of hairless rat skin

There was a statistical difference between the groups in the one-way ANOVA test for 8-OHdG positivity (df = 5, *F* = 51.293). In the sections of pancreatic tissue from the control and EGCG groups, the endocrine cells in the islets of Langerhans and acinar epithelial cells in the exocrine pancreas were immune-negative (Fig. 8A, B; Table 8, *p* = 0.001, 8-OHdG positivity scores 0 (0–0) and 0 (0–1)). In the CDDP group, we determined extensive immune positivity in endocrine cells in the islets of Langerhans and exocrine acinar epithelial cells for the *8-OHdG* primary antibody (Fig. 8C; Table 8, *p* = 0.001, 8-OHdG positivity score 2 (2–3)). In contrast, in the CDDP + EGCG, CDDP + INF, and CDDP + EGCG + INF groups, we observed fewer endocrine cells showing 8-OHdG positivity (Fig. 8D–F; Table 8, *p* = 0.001, 8-OHdG positivity scores 0 (0–1), 0 (0–1), and 0 (0–1), respectively).

## Discussion

The present study has shown that cisplatin causes damage to pancreatic tissues and pancreatic islets of Langerhans cells by increasing oxidative stress and inducing apoptosis. On the other hand, EGCG and infliximab exerted protective effects on pancreatic tissues and pancreatic islets of Langerhans cells when administered alone and in combination. Although the histological investigation revealed a slightly enhanced protective effect when EGCG and infliximab were used together, the biochemical and immunohistochemical analyses yielded similar results.

Cisplatin toxicity is associated with oxidative stress and the production of reactive oxygen species (ROS), as well as elevated release of proinflammatory cytokines such as TNF-α and interleukin-6 (IL-6) [3]. This causes cellular DNA damage and, consequently, apoptosis [2]. Pancreatic β-cell damage and apoptosis lead to a decrease in pancreatic mass, ultimately developing hyperglycemia [8, 40]. There are case reports regarding the development of acute hyperglycemia in patients receiving CDDP treatment [10]. Moreover, individuals with a history of CDDP treatment were reported to have a higher risk of diabetes mellitus (DM) compared to matched control groups [41]. The reduction of inflammatory cytokines and ROS production may protect healthy tissues from CDDP-induced damage [3]. As in our study, many studies have shown that CDDP increases TNF-α and nuclear factor kappa B (NF-κB) levels through oxidative stress [7, 8]. Meanwhile, the administration of EGCG and/or infliximab before CDDP chemotherapy alleviated inflammation and apoptosis via the reduction of TNF-α levels.

Insulin and glucagon-producing alpha and beta cells in the islets of Langerhans in CDDP-treated rat pancreatic tissue showed decreased immunopositivity. The decreased immunopositivity of alpha and beta cells can be explained in two different ways. First, these cells may have been stained immune-negative due to the inactive state of these cells. In this case, the cells stop secreting insulin or glucagon for the moment, so they stain immune-negative but are alive. Secondly, the alpha and beta cells may be immuno-negative because apoptosis or necroptosis has occurred, and the number of cells has decreased considerably. In order to clarify this situation, we performed TUNEL staining, which is an indicator of apoptosis, and showed that TUNEL positivity and, therefore, apoptosis increased in immune-negative stained cells. Therefore, we think that insulin and glucagon-secreting beta and alpha cells are immune-negative because they undergo apoptosis. In addition, vacuolization accompanied by loss of cytoplasm in our histopathological examinations strengthens the possibility of apoptosis.

The primary goal with respect to the prevention of diabetes is the protection of the function of β-cells found in the islets of Langerhans in the pancreas [42]. Pancreatic islets are negatively affected by oxygen-free radicals and inflammatory cytokines [43]. A protective shield with anti-oxidative and anti-inflammatory effects may be needed to preserve their viability. EGCG is a polyphenol that is found to be most abundant in green tea and is the most beneficial of all catechins [20]. Polyphenols were shown to be safe for humans with protective effects against DNA damage and ROS [16]. Accordingly, EGCG was shown to possess antioxidant and anti-inflammatory effects in both human and animal studies [44]. In addition to the prevention of free radical damage, cancer prevention, and anti-oxidative effects, EGCG also has a therapeutic impact on glucose and lipid metabolism [45]. β-cells are known to be more susceptible to oxidative damage and apoptosis compared to other cells [46]. Faheem and Ali showed that EGCG had a potential protective effect against pancreatic damage related to immobilization stress in a rat model [47]. In this line, the comparison is done with the earlier study, and the present study is in line with it, providing a decrease in MDA and TNF-α levels while increasing GSH levels. On the other hand, the immune negativity seen in TUNEL and 8-OHdG suggests that EGCG lowers cell damage and apoptosis by reducing inflammation and its antioxidant effects. Hara et al. demonstrated that EGCG protected islet mass and β-cell function by preventing the increase in 8-OHdG in the F344 rat pancreas [48]. Dickinson et al. reported that EGCG improved insulin sensitivity and β-cell function by scavenging free radicals as well as increasing the levels of DNA repair proteins and antioxidants in diabetic mice [16]. Cao et al. showed that EGCG suppressed inflammation by reducing TNF-α, partially reversed metabolically abnormalities, and increased insulin sensitivity by preserving pancreatic histology in rats fed with a high-fat diet [21]. A study conducted by Wada et al. found that EGCG preserved the size and function of pancreatic islets in mice by upregulating the production of antioxidant enzymes [49]. This supports the findings of the present investigation.

Previous studies have reported that supraphysiological doses of EGCG could induce apoptosis and autophagy [16, 49]. While low concentrations of EGCG decrease ROS production, high doses may significantly increase ROS production; therefore, it is crucial to identify the most effective dose without side effects. Based on our literature review, we administered EGCG via the i.p. route at a dose that was determined to be safe with a low side effect profile [28]. Accordingly, we considered 50 mg/kg/day EGCG the most appropriate concentration for the survival of islet cells.

Consistent with the results of our study, studies have shown EGCG to decrease TNF-α expression in addition to reducing oxidative stress [21]. As well as reducing the lipid profile and oxidative stress, EGCG improved glycemic control, insulin sensitivity, and β-cell function more than metformin in studies on rat models of type 2 DM [42]. In a mice study by Ren et al., EGCG provided glycemic control by significantly increasing c-peptide levels [45]. Wu et al. found that EGCG protected pancreatic β-cells against ethanol-induced endoplasmic reticulum stress and oxidative damage-induced apoptosis [50]. EGCG improved the oxidative damage of iron-loaded β-cells by removing redox iron and free radicals in insulinoma pancreatic β-cells in rats exposed to redox iron [51]. This may indicate that EGCG has a cytoprotective effect on β-cells and reduces apoptosis through an anti-inflammatory and antioxidant effect.

Pancreatic islet cells are highly sensitive to oxidative stress due to their high secretory functions and low antioxidant capacity [52]. The increased cellular damage and apoptosis due to oxidative stress caused by cisplatin lead to lipid peroxidation in cell membranes. MDA is the end product of membrane lipid peroxidation [47]. MDA levels increased in pancreatic tissues of cisplatin-administered rats, indicating a significant increase in lipid peroxidation. The low MDA levels in rats administered EGCG may be an indication that EGCG reduces DNA damage and apoptosis caused by ROS. Glutathione is an essential nonenzymatic endogenous antioxidant [53]. In cases of extreme oxidative stress, GSH levels decrease due to increased use [53]. GSH levels in the pancreas of cisplatin-treated rats were low, probably due to increased excessive oxidative stress. We think that EGCG reduces oxidative stress in rat pancreatic tissue, resulting in less GSH consumption. Infliximab also prevented membrane lipid peroxidation in rat pancreatic tissue with anti-inflammatory and antioxidant capacity similar to EGCG and also reduced the depletion of GSH levels. Co-administration of EGCG and infliximab did not show a synergistic effect. This may be because inflammation and oxidative stress in rat pancreatic tissue were already sufficiently suppressed by the administration of each agent alone. Furthermore, neither EGCG nor infliximab directly targets the function and cellular structure of alpha and beta cells. Instead, their main purpose is to inhibit oxidative stress and inflammation in a broader sense. Since both agents alone have sufficiently suppressed oxidative stress and inflammation, there may not be a damaged environment where they can exert greater efficacy when used together. This situation can be likened to the fact that N-acetylcysteine does not show an increasing effect on GSH levels in healthy conditions without oxidative stress [53].

TNF-α, which is an inflammatory cytokine produced by macrophages and monocytes, is effective in the development of various diseases as well as cancer and can also result in the development of DM by causing pancreatic β-cells to undergo apoptosis [54, 55]. TNF-α is among the main mediators that trigger inflammation in pancreatic islets of Langerhans, which increases apoptosis among β-cells by activating NF-κB [56, 57]. Thus, the use of TNF-α inhibitors in the treatment of diabetes appears promising [58, 59]. Results obtained from observational studies indicate that the use of TNF-α inhibitors in rheumatoid arthritis (RA) patients is associated with a lower DM incidence and better metabolic control compared to non-biological agents [60, 61]. Anti-TNF treatment achieved normoglycemia and positively affected metabolic parameters in numerous human studies and animal models of type 1 DM [62–64]. TNF-α inhibitors were reported to have positive effects on diabetic peripheral neuropathy (DPN) and diabetic nephropathy in diabetic animal models [65]. Similarly, Quattrin et al. demonstrated a protective effect on β-cells by golimumab, which is an anti-TNF-α agent, in those with newly diagnosed type 1 diabetes [66]. Golimumab treatment promoted endogenous insulin production in children and adolescents with newly diagnosed type 1 diabetes, while the need for exogenous insulin declined [66]. Anti-TNF agents have been shown to treat inflammatory diseases by inhibiting TNF-α-mediated inflammation and ROS [19]. In a study by Gómez-Hernández et al., a 52-week anti-TNF treatment was able to reduce NF-κB activation in BATIRKO mice [67]. Infliximab is known to inhibit TNF-α and reduce proinflammatory cytokine levels in the microenvironment [18, 19]. Hence, this reduces tissue damage by blocking the direct effects of cytokines and their stimulation by oxidative stress [6]. The present study shows that infliximab reduces TNF-α and MDA levels while increasing tissue GSH levels. In support of our results, studies show that TNF-α blockage is required in order to achieve significant protection against inflammatory damage and enhance the outcomes in those who underwent pancreatic islet autotransplantation [54].

In the present study, we focused not only on β-cells but also on α-cells in evaluating the effects of EGCG and infliximab on pancreatic islets. Because just as β-cell function is important for the prevention of hyperglycemia, α-cell function is important for the prevention of hypoglycemia [20]. Adequate glucagon secretion from α-cells is needed so that hypoglycemia does not occur [68]. In a study on a pancreatic cell line, Cao et al. showed that EGCG protected α-cell function through its antioxidant effects [20]. Similarly, in the present study, both EGCG and infliximab also exerted an antioxidant effect on α-cells, increasing cell survival and decreasing apoptosis.

Concerns regarding the tumor-protective effects of the available agents represent the greatest obstacle to the use of antioxidants to reduce cancer treatment–induced damage in healthy tissues. We had the same concerns in the present study. However, polyphenols are potent anti-tumor agents that can play a role against cisplatin-induced damage as natural products [3]. EGCG was reported to reduce tumor weight by functioning as an IGF1R antagonist in cancer cells [69]. Moreover, EGCG was shown to serve as a tumor suppressor and induce apoptosis in cancer cells in hepatocellular carcinoma [70]. On the other hand, no cases of increasing cancer prevalence or tumor progression have been reported with the use of infliximab [71, 72]. On the contrary, there is evidence suggesting that high TNF-α levels are associated with harmful effects such as cancer cell growth, invasion, and metastasis [72, 73]. Furthermore, there is a study in the literature in which high TNF-α levels in colon cancer resulted in oxaliplatin resistance, which was prevented by infliximab [73].

Our study is an exploratory pilot study that investigates the effects of EGCG and infliximab on CDDP-induced pancreatic tissue damage both in combination and separately. However, this study should be interpreted in consideration of certain limitations. The primary and most important limitation is that we did not determine the tumor-protective effects of EGCG or infliximab. Therefore, it will be reasonable to investigate the effects of both EGCG and infliximab on tumor cells, primarily on cancer cell cultures, in the future. Another important limitation that should be considered is that the results of this study exhibit short-term effects. Long-term studies are needed to determine the chronic effects. The present study is also an animal model. Human studies are warranted so that they can be applied to clinical use. First of all, the effective and safe dose ranges and administration frequency of the EGCG should be determined in human studies. In the present study, EGCG was administered for 3 days and at a fixed dosage, and future studies must compare different doses and frequencies to determine the most effective safety dose and frequency of administration. Furthermore, future studies comparing the protective effects of EGCG and infliximab with other commercially available drugs are needed. It is known that EGCG and infliximab exert an anti-inflammatory effect by suppressing TNF-α. However, the detailed mechanisms of the protective effect of both are unknown. In future studies, the mechanisms of this protective effect of both agents should be investigated in detail. It would also be useful to measure the levels of amylase, lipase, glucose, insulin, c-peptide, HbA1c, and glucagon biochemically and detect wet pancreas weights to determine edema.

Oxidative stress is thought to be one of the factors that cause DM development by causing beta cell damage [54]. Before the development of type 2 DM, there is an irreversible chronic process called prediabetes [74]. In this process, preventing or reversing pancreatic islet cell damage may prevent the development of DM. After determining the effective and safe dose of EGCG in future studies, the capacity of long-term administration in the prediabetic period to prevent the development of DM can be determined by animal and human studies. Literature studies are showing that anti-TNF therapy has protective effects on beta cells in type 1 DM and pancreatic islet cell transplantation [63, 66]. Type 1 DM development is not predictable. However, in future studies, it may be possible to determine the preventive effect of administering infliximab or EGCG to animal models in which insulin and antibodies against islet cells are injected.

## Conclusion

In summary, this study shows that cisplatin has an acute effect on pancreatic tissue and islet cells that increases oxidation and apoptosis. The administration of EGCG and infliximab, individually and in combination, may alleviate cisplatin-induced pancreatic injury, at least in the acute period.

## Data Availability

Data will be made available on request.

## Abbreviations

8-OHdG: 8-hydroxy-2′-deoxyguanosine
CDDP: Cisplatin, cis-diamminedichloroplatinum
DM: Diabetes mellitus
EGCG: Epigallocatechin-3-gallate
GSH: Glutathione
HDS: Histopathological damage score
IARC: International Agency for Research on Cancer
IL-6: Interleukin-6
INF: Infliximab
i.p.: Intraperitoneal
MDA: Malondialdehyde
NF-κB: Nuclear factor kappa B
ROS: Reactive oxygen species
TBARS: Thiobarbituric acid reactive substances
TBA: Thiobarbituric acid
TNF-α: Tumor necrosis factor-alpha
TT: Total thiol
TUNEL: Terminal deoxynucleotidyl transferase dUTP-mediated nick end labeling

## References

[1] Dasari S, Bernard Tchounwou P (2014) Cisplatin in cancer therapy: molecular mechanisms of action. Eur J Pharmacol 740:364–378. 10.1016/j.ejphar.2014.07.025 25058905 10.1016/j.ejphar.2014.07.025 PMC4146684

[2] Ghosh S (2019) Cisplatin: the first metal based anticancer drug. Bioorg Chem 88:102925. 10.1016/j.bioorg.2019.102925 31003078 10.1016/j.bioorg.2019.102925

[3] Dasari S, Njiki S, Mbemi A et al (2022) Pharmacological effects of Cisplatin Combination with Natural products in Cancer Chemotherapy. Int J Mol Sci 23:1–25. 10.3390/ijms23031532 10.3390/ijms23031532 PMC8835907 35163459

[4] Mercantepe F, Mercantepe T, Topcu A et al (2018) Protective effects of amifostine, curcumin, and melatonin against cisplatin-induced acute kidney injury. Naunyn Schmiedebergs Arch Pharmacol. 10.1007/s00210-018-1514-4 10.1007/s00210-018-1514-4 29860655

[5] Yin H, Zhang H, Kong Y et al (2020) Apelin protects auditory cells from cisplatin-induced toxicity in vitro by inhibiting ROS and apoptosis. Neurosci Lett 728:134948. 10.1016/j.neulet.2020.134948 32278025 10.1016/j.neulet.2020.134948

[6] Cumhur Cüre M, Cüre E, Kalkan Y et al (2016) Infliximab modulates cisplatin-induced hepatotoxicity in rats. Balkan Med J 33:504–511. 10.5152/balkanmedj.2016.150576 27761277 10.5152/balkanmedj.2016.150576 PMC5056652

[7] Bakir M, Geyikoglu F, Koç K, Cerig S (2018) Therapeutic effects of oleuropein on cisplatin–induced pancreas injury in rats ABSTRACT. J Cancer Res Ther 14:671–678. 10.4103/jcrt.JCRT 29893338 10.4103/jcrt.JCRT_1040_16

[8] Stošić B, Janković R, Stošić M et al (2020) Caffeic acid phenethyl ester attenuates changes in pancreatic tissue damage biomarkers induced by cisplatin. Can J Physiol Pharmacol 98:296–303. 10.1139/cjpp-2019-0374 31825661 10.1139/cjpp-2019-0374

[9] Yadav YC (2019) Effect of cisplatin on pancreas and testies in Wistar rats: biochemical parameters and histology. Heliyon 5:e02247. 10.1016/j.heliyon.2019.e02247 31453403 10.1016/j.heliyon.2019.e02247 PMC6700420

[10] Nan DN, Fernández-Ayala M, Vega Villegas ME et al (2003) Diabetes mellitus following cisplatin treatment. Acta Oncol (Madr) 42:75–78. 10.1080/0891060310002276 10.1080/0891060310002276 12665335

[11] Zhou J, Nie RC, Yin YX et al (2022) Protective effect of Natural antioxidants on reducing Cisplatin-Induced Nephrotoxicity. Dis Markers 2022. 10.1155/2022/1612348 10.1155/2022/1612348 PMC9678481 36419843

[12] Lopez AJ, Lau H, Li S, Ichii H (2020) Potential benefits of nrf2/keap1 targeting in pancreatic islet cell transplantation. Antioxidants 9:1–12. 10.3390/antiox9040321 10.3390/antiox9040321 PMC7222398 32316115

[13] Tekin C, Aberson HL, Bijlsma MF, Spek CA (2020) Early macrophage infiltrates impair pancreatic cancer cell growth by TNF-α secretion. BMC Cancer 20:1–9. 10.1186/s12885-020-07697-1 10.1186/s12885-020-07697-1 PMC7709323 33267818

[14] Younis NN, Mohamed HE, Shaheen MA et al (2021) Inactivation of Wnt/β-catenin/renin angiotensin axis by tumor necrosis factor-alpha inhibitor, infliximab, ameliorates CKD induced in rats. Biochem Pharmacol 185:114426. 10.1016/j.bcp.2021.114426 33482150 10.1016/j.bcp.2021.114426

[15] Wood PR, Manning E, Baker JF et al (2018) Blood glucose changes surrounding initiation of tumor-necrosis factor inhibitors and conventional disease-modifying anti-rheumatic drugs in veterans with rheumatoid arthritis. World J Diabetes 9:53–58. 10.4239/wjd.v9.i2.53 29531640 10.4239/wjd.v9.i2.53 PMC5840570

[16] Dickinson D, Derossi S, Yu H et al (2015) Epigallocatechin-3-gallate modulates antioxidant defense enzyme expression in murine submandibular and pancreatic exocrine gland cells and human HSG cells. Autoimmunity 47:177–184. 10.3109/08916934.2013.879470.Epigallocatechin-3-gallate 10.3109/08916934.2013.879470 PMC4301570 24444391

[17] Sanchez-Hernandez JG, Rebollo N, Munoz F et al (2019) Therapeutic drug monitoring of tumour necrosis factor inhibitors in the management of chronic inflammatory diseases. Ann Clin Biochem 56:28–41. 10.1177/0004563218782286 29807436 10.1177/0004563218782286

[18] Melsheimer R, Geldhof A, Apaolaza I, Schaible T (2019) Remicade® (Infliximab): 20 years of contributions to science and medicine. Biol Targets Ther 13:139–178. 10.2147/BTT.S207246 10.2147/BTT.S207246 PMC6679695 31440029

[19] Leone GM, Mangano K, Petralia MC et al (2023) Past, Present and (foreseeable) future of Biological Anti-TNF alpha therapy. J Clin Med 12. 10.3390/jcm12041630 10.3390/jcm12041630 PMC9963154 36836166

[20] Cao T, Zhang X, Yang D et al (2018) Antioxidant effects of epigallocatechin-3-gallate on the aTC1-6 pancreatic alpha cell line. Biochem Biophys Res Commun 495:693–699. 10.1016/j.bbrc.2017.11.006 29117537 10.1016/j.bbrc.2017.11.006

[21] Cao Y, Bao S, Yang W et al (2014) Epigallocatechin gallate prevents inflammation by reducing macrophage infiltration and inhibiting tumor necrosis factor-α signaling in the pancreas of rats on a high-fat diet. Nutr Res 34:1066–1074. 10.1016/j.nutres.2014.10.004 25453543 10.1016/j.nutres.2014.10.004

[22] Wu D, Liu Z, Li J et al (2019) Epigallocatechin-3-gallate inhibits the growth and increases the apoptosis of human thyroid carcinoma cells through suppression of EGFR/RAS/RAF/MEK/ERK signaling pathway. Cancer Cell Int 19:1–17. 10.1186/s12935-019-0762-9 30858760 10.1186/s12935-019-0762-9 PMC6394055

[23] Khiewkamrop P, Surangkul D, Srikummool M et al (2022) Epigallocatechin gallate triggers apoptosis by suppressing de novo lipogenesis in colorectal carcinoma cells. FEBS Open Bio 12:937–958. 10.1002/2211-5463.13391 35243817 10.1002/2211-5463.13391 PMC9063442

[24] du Sert NP, Ahluwalia A, Alam S et al (2020) Reporting animal research: explanation and elaboration for the arrive guidelines 2.0. BMC Vet Res 16:1–7. 10.1186/s12917-020-02451-y 32660541 10.1186/s12917-020-02451-y PMC7359286

[25] Arifin WN, Zahiruddin WM (2017) Sample size calculation in animal studies using resource equation approach. Malaysian J Med Sci 24:101–105. 10.21315/mjms2017.24.5.11 10.21315/mjms2017.24.5.11 PMC5772820 29386977

[26] Johnson PD, Besselsen DG (2002) Practical aspects of experimental design in animal research. ILAR J 43:202–206. 10.1093/ilar.43.4.202 12391395 10.1093/ilar.43.4.202

[27] Charan J, Kantharia N (2013) How to calculate sample size in animal studies? J Pharmacol Pharmacother 4:303–306. 10.4103/0976-500X.119726 24250214 10.4103/0976-500X.119726 PMC3826013

[28] Ramachandran B, Jayavelu S, Murhekar K, Rajkumar T (2016) Repeated dose studies with pure Epigallocatechin-3-gallate demonstrated dose and route dependant hepatotoxicity with associated dyslipidemia. Toxicol Rep 3:336–345. 10.1016/j.toxrep.2016.03.001 28959554 10.1016/j.toxrep.2016.03.001 PMC5615837

[29] El-Missiry MA, Othman AI, El-Sawy MR, Lebede MF (2018) Neuroprotective effect of epigallocatechin-3-gallate (EGCG) on radiation-induced damage and apoptosis in the rat hippocampus. Int J Radiat Biol 94:798–808. 10.1080/09553002.2018.1492755 29939076 10.1080/09553002.2018.1492755

[30] Altintas N, Erboga M, Aktas C et al (2016) Protective effect of Infliximab, a Tumor necrosis factor-alfa inhibitor, on Bleomycin-Induced lung fibrosis in rats. Inflammation 39:65–78. 10.1007/s10753-015-0224-z 26253295 10.1007/s10753-015-0224-z

[31] El-shafaei A, Abdelmaksoud R, Elshorbagy A et al (2018) Protective effect of melatonin versus montelukast in cisplatin-induced seminiferous tubule damage in rats. Andrologia 50:1–8. 10.1111/and.13077 10.1111/and.13077 30019386

[32] Jahan S, Munawar A, Razak S et al (2018) Ameliorative effects of rutin against cisplatin-induced reproductive toxicity in male rats. BMC Urol 18:1–11. 10.1186/s12894-018-0421-9 30463555 10.1186/s12894-018-0421-9 PMC6249881

[33] Flecknell P, Lofgren JLS, Dyson MC et al (2015) Preanesthesia, anesthesia, analgesia, and euthanasia. American College of Laboratory Animal Medicine 2015:1135–1200. 10.1016/B978-0-12-409527-4.00024-9

[34] Ohkawa H, Ohishi N, Yagi K (1979) Assay for lipid peroxides in animal tissues by thiobarbituric acid reaction. Anal Biochem 95:351–358. 10.1016/0003-2697(79)90738-3 36810 10.1016/0003-2697(79)90738-3

[35] Sedlak J, Lindsay RH (1968) Estimation of total, protein-bound, and nonprotein sulfhydryl groups in tissue with Ellman’s reagent. Anal Biochem 25:192–205. 10.1016/0003-2697(68)90092-4 4973948 10.1016/0003-2697(68)90092-4

[36] Schmidt J, Rattner DW, Lewandrowski K et al (1992) A better model of acute pancreatitis for evaluating therapy. Ann Surg 215:44–56. 10.1097/00000658-199201000-00007 1731649 10.1097/00000658-199201000-00007 PMC1242369

[37] Fedchenko N, Reifenrath J (2014) Different approaches for interpretation and reporting of immunohistochemistry analysis results in the bone tissue - a review. Diagn Pathol 9:221. 10.1186/s13000-014-0221-9 25432701 10.1186/s13000-014-0221-9 PMC4260254

[38] El Agaty M, Ibrahim Ahmed S A (2020) Pathophysiological and immunohistochemical analysis of pancreas after renal ischemia/reperfusion injury: protective role of melatonin*. Arch Physiol Biochem 126:264–275. 10.1080/13813455.2018.1517182 30270672 10.1080/13813455.2018.1517182

[39] O’Brien TD, Hayden DW, Johnson KH, Fletcher TF (1986) Immunohistochemical morphometry of pancreatic endocrine cells in diabetic, normoglycaemic glucose-intolerant and normal cats. J Comp Pathol 96:357–369. 10.1016/0021-9975(86)90031-9 2874160 10.1016/0021-9975(86)90031-9

[40] Liu H, Wang L, Li F et al (2021) The synergistic protection of EGCG and quercetin against streptozotocin (STZ)-induced NIT-1 pancreatic β cell damage via upregulation of BCL-2 expression by miR-16-5p. J Nutr Biochem 96:108748. 10.1016/j.jnutbio.2021.108748 34051305 10.1016/j.jnutbio.2021.108748

[41] Nguyen NAMP, Vos P, Vinh-hung V et al (2009) Altered glucose metabolism during chemoradiation for Head and Neck Cancer. Anticancer Res 29:4683–4687 20032420

[42] Zhu T, Li M, Zhu M et al (2022) Epigallocatechin-3-gallate alleviates type 2 diabetes mellitus via β-cell function improvement and insulin resistance reduction. Iran J Basic Med Sci 25:483–488. 10.22038/IJBMS.2022.58591.13016 35656076 10.22038/IJBMS.2022.58591.13016 PMC9150804

[43] Mercantepe F, Tumkaya L, Mercantepe T et al (2023) Radioprotective effects of α2-adrenergic receptor agonist dexmedetomidine on X-ray irradiation-induced pancreatic islet cell damage. Naunyn Schmiedebergs Arch Pharmacol. 10.1007/s00210-023-02454-0 36877270 10.1007/s00210-023-02454-0

[44] Pournourmohammadi S, Grimaldi M, Stridh MH et al (2017) Epigallocatechin-3-gallate (EGCG) activates AMPK through the inhibition of glutamate dehydrogenase in muscle and pancreatic ß-cells: a potential beneficial effect in the pre-diabetic state? Int J Biochem Cell Biol 88:220–225. 10.1016/j.biocel.2017.01.012 28137482 10.1016/j.biocel.2017.01.012

[45] Ren Z, Yang Z, Lu YI et al (2020) Anti – glycolipid disorder effect of epigallocatechin – 3 – gallate on high – fat diet and STZ – induced T2DM in mice. Mol Med Rep 21:2475–2483. 10.3892/mmr.2020.11041 32236613 10.3892/mmr.2020.11041 PMC7185284

[46] Li W, Zhu C, Liu T et al (2020) Epigallocatechin-3-gallate ameliorates glucolipid metabolism and oxidative stress in type 2 diabetic rats. Diabetes Vasc Dis Res 17. 10.1177/1479164120966998 10.1177/1479164120966998 PMC7919214 33280417

[47] Faheem NM, Ali TM (2021) The counteracting effects of (-)-Epigallocatechin-3-Gallate on the immobilization stress-induced adverse reactions in rat pancreas. Cell Stress Chaperones 26:159–172. 10.1007/s12192-020-01165-2 33000400 10.1007/s12192-020-01165-2 PMC7736449

[48] Hara Y, Fujino M, Takeuchi M, Li X (2007) Green-tea polyphenol (-)-epigallocatechin-3-gallate provides resistance to apoptosis in isolated islets. J Hepatobiliary Pancreat Surg 14:493–497. 10.1007/s00534-006-1207-0 17909719 10.1007/s00534-006-1207-0

[49] Wada Y, Takata A, Ikemoto T et al (2019) The protective effect of epigallocatechin 3-gallate on mouse pancreatic islets via the Nrf2 pathway. Surg Today 49:536–545. 10.1007/s00595-019-1761-0 30730004 10.1007/s00595-019-1761-0

[50] Wu T, Xiang J, Shan W et al (2016) Epigallocatechin-3-Gallate inhibits ethanol-Induced apoptosis through Neurod1 regulating CHOP expression in pancreatic β-Cells. Anat Rec 299:573–582. 10.1002/ar.23332 10.1002/ar.23332 26916663

[51] Koonyosying P, Uthaipibull C, Fucharoen S et al (2019) Decrement in Cellular Iron and reactive oxygen species, and improvement of insulin secretion in a pancreatic cell line using Green Tea Extract. Pancreas 48:636–643. 10.1097/MPA.0000000000001320 31091209 10.1097/MPA.0000000000001320 PMC6553981

[52] Moens C, Muller CJF, Bouwens L (2022) In vitro comparison of various antioxidants and flavonoids from Rooibos as beta cell protectants against lipotoxicity and oxidative stress-induced cell death. PLoS ONE 17:1–16. 10.1371/journal.pone.0268551 10.1371/journal.pone.0268551 PMC9113568 35580081

[53] Rushworth GF, Megson IL (2014) Existing and potential therapeutic uses for N-acetylcysteine: the need for conversion to intracellular glutathione for antioxidant benefits. Pharmacol Ther 141:150–159. 10.1016/j.pharmthera.2013.09.006 24080471 10.1016/j.pharmthera.2013.09.006

[54] Burke SJ, Collier JJ (2021) Special issue: Islet inflammation and metabolic homeostasis. Metabolites 11:77. 10.3390/metabo11020077 10.3390/metabo11020077 PMC7910950 33525362

[55] Degen AS, Krynytska IY, Kamyshnyi AM (2020) Changes in the transcriptional activity of the entero-insular axis genes in streptozotocin-induced diabetes and after the administration of TNF-α non-selective blockers. Endocr Regul 54:160–171. 10.2478/enr-2020-0019 32857721 10.2478/enr-2020-0019

[56] Akash MSH, Rehman K, Liaqat A (2018) Tumor necrosis Factor-Alpha: role in development of insulin resistance and Pathogenesis of type 2 diabetes Mellitus. J Cell Biochem 119:105–110. 10.1002/jcb.26174 28569437 10.1002/jcb.26174

[57] Contreras CJ, Mukherjee N, Branco RCS et al (2022) RIPK1 and RIPK3 regulate TNFα-induced β-cell death in concert with caspase activity. Mol Metab 65:101582. 10.1016/j.molmet.2022.101582 36030035 10.1016/j.molmet.2022.101582 PMC9464965

[58] Galligan A, Krishnamurthy B, Kay TW (2020) Successful treatment of immune checkpoint inhibitor-induced diabetes with infliximab. Diabetes Care 42:e153–e154. 10.2337/dc19-1747 10.2337/dc19-1747 31862826

[59] Trinh B, Donath MY, Läubli H (2020) Successful treatment of immune checkpoint inhibitor-induced diabetes with infliximab. Diabetes Care 42:e153–e154. 10.2337/dci19-0058 10.2337/dc19-0908 31308021

[60] Desai RJ, Dejene S, Jin Y et al (2020) Comparative risk of diabetes Mellitus in patients with rheumatoid arthritis treated with biologic or targeted synthetic disease-modifying drugs: a Cohort Study. ACR Open Rheumatol 2:222–231. 10.1002/acr2.11124 32267094 10.1002/acr2.11124 PMC7164631

[61] Bissell LA, Hensor EMA, Kozera L et al (2016) Improvement in insulin resistance is greater when infliximab is added to methotrexate during intensive treatment of early rheumatoid arthritis-results from the IDEA study. Rheumatol (United Kingdom) 55:2181–2190. 10.1093/rheumatology/kew306 10.1093/rheumatology/kew306 27638812

[62] Danve AS, Kulkarni S (2017) Do tumor necrosis factor (TNF) inhibitors improve the glycemic control in patients with rheumatoid arthritis and concomitant diabetes mellitus? Am J Ther 24:e347–e350. 10.1097/MJT.0000000000000297 26103543 10.1097/MJT.0000000000000297

[63] Baskal S, Tsikas SA, Begou O et al (2022) Advanced Glycation End-products (AGEs) of lysine and effects of Anti-TCR/Anti-TNF-α antibody-based therapy in the LEW.1AR1-iddm rat, an animal model of human type 1 diabetes. Int J Mol Sci 23. 10.3390/ijms23031541 10.3390/ijms23031541 PMC8915180 35163462

[64] Bollenbach A, Tsikas D, Lenzen S, Jörns A (2020) Asymmetric dimethylation and citrullination in the LEW.1AR1-iddm rat, an animal model of human type 1 diabetes, and effects of anti-TCR/anti-TNF-α antibody-based therapy. Amino Acids 52:103–110. 10.1007/s00726-019-02811-5 31832896 10.1007/s00726-019-02811-5

[65] Maekawa M, Tadaki H, Tomimoto D et al (2019) A novel TNF-α converting enzyme (TACE) selective inhibitor JTP-96193 prevents insulin resistance in KK-Ay type 2 diabetic mice and diabetic peripheral neuropathy in type 1 diabetic mice. Biol Pharm Bull 42:1906–1912. 10.1248/bpb.b19-00526 31685773 10.1248/bpb.b19-00526

[66] Quattrin T, Haller MJ, Steck AK et al (2020) Golimumab and Beta-cell function in Youth with New-Onset type 1 diabetes. N Engl J Med 383:2007–2017. 10.1056/nejmoa2006136 33207093 10.1056/NEJMoa2006136

[67] Gómez-Hernández A, Perdomo L, de las Heras N et al (2014) Antagonistic effect of TNF-alpha and insulin on uncoupling protein 2 (UCP-2) expression and vascular damage. Cardiovasc Diabetol 13:4–13. 10.1186/s12933-014-0108-9 25077985 10.1186/s12933-014-0108-9 PMC4149264

[68] Braun M (2014) The somatostatin receptor in human pancreatic β-cells. Vitam Horm 95:165–93. 10.1016/B978-0-12-800174-5.00007-7 24559918 10.1016/B978-0-12-800174-5.00007-7

[69] Duan G, Tang Q, Yan H et al (2017) A strategy to Delay the Development of Cisplatin Resistance by maintaining a certain amount of cisplatin-sensitive cells. Sci Rep 7:1–13. 10.1038/s41598-017-00422-2 28348367 10.1038/s41598-017-00422-2 PMC5428423

[70] Kuo P-L, Lin C-C (2003) Biomedical Science Green Tea Constituent (-) -Epigallocatechin- 3-Gallate inhibits hep G2 cell proliferation and induces apoptosis through p53-Dependent and Fas-mediated pathways. Orig Pap J Biomed Sci 10:219–227. 10.1159/000068711 10.1007/BF02256057 12595758

[71] Perdigoto AL, Deng S, Du KC et al (2022) Immune cells and their inflammatory mediators modify β cells and cause checkpoint inhibitor–induced diabetes. JCI Insight 7:10–14. 10.1172/jci.insight.156330 10.1172/jci.insight.156330 PMC9536276 35925682

[72] Mercogliano MF, Bruni S, Elizalde PV, Schillaci R (2020) Tumor necrosis factor α blockade: an opportunity to tackle breast Cancer. Front Oncol 10. 10.3389/fonc.2020.00584 10.3389/fonc.2020.00584 PMC7189060 32391269

[73] Huang D, Xue J, Li S, Yang D (2018) Oxaliplatin and infliximab synergize to induce regression of colon cancer. Oncol Lett 15:1517–1522. 10.3892/ol.2017.7468 29434844 10.3892/ol.2017.7468 PMC5774490

[74] Klisic A, Kavaric N, Stanisic V et al (2020) Endocan and a novel score for dyslipidemia, oxidative stress and inflammation (DOI score) are independently correlated with glycated hemoglobin (HbA1c) in patients with prediabetes and type 2 diabetes. Arch Med Sci 16:42–50. 10.5114/aoms.2019.87541 32051704 10.5114/aoms.2019.87541 PMC6963142

